# Enhanced photocatalytic and antibacterial activities of novel Ag-HA bioceramic nanocatalyst for waste-water treatment

**DOI:** 10.1038/s41598-023-40970-4

**Published:** 2023-08-24

**Authors:** Sherif Elbasuney, Ahmed M. El-Khawaga, Mohamed A. Elsayed, Amir Elsaidy, Miguel A. Correa-Duarte

**Affiliations:** 1 Military Technical College, Egyptian Armed Forces, Cairo, Egypt; 2 School of Chemical Engineering, Military Technical College, Cairo, Egypt; 3 Department of Basic Medical Sciences, Faculty of Medicine, Galala University, New Galala City, Suez, Egypt; 4 Department of Physical Chemistry, Biomedical Research Center (CINBIO), and Institute of Biomedical Research of Ourense-Pontevedra-Vigo (IBI), Universidad de Vigo, 36310 Vigo, Spain

**Keywords:** Nanoscience and technology, Environmental chemistry

## Abstract

Hydroxyapatite (HA), the most common bioceramic material, offers attractive properties as a catalyst support. Highly crystalline mono-dispersed silver doped hydroxyapatite (Ag-HA) nanorods of 60 nm length was developed via hydrothermal processing. Silver dopant offered enhanced chemisorption for crystal violet (CV) contaminant. Silver was found to intensify negative charge on the catalyst surface; in this regard enhanced chemisorption of positively charged contaminants was accomplished. Silver dopant experienced decrease in the binding energy of valence electron for oxygen, calcium, and phosphorous using X-ray photoelectron spectroscopy XPS/ESCA; this finding could promote electron–hole generation and light absorption. Removal efficiency of Ag-HA nanocomposite for CV reached 88% after the synergistic effect with 1.0 mM H_2_O_2_; silver dopant could initiate H_2_O_2_ cleavage and intensify the release of active ȮH radicals. Whereas HA suffers from lack of microbial resistance; Ag-HA nanocomposite demonstrated high activity against Gram-positive (*S. aureus*) bacteria with zone of inhibition (ZOI) mm value of 18.0 mm**,** and high biofilm inhibition of 91.1%. Ag-HA nanocompsite experienced distinctive characerisitcs for utilization as green bioceramic photocatalyst for wastewater treatment.

## Introduction

Difficulties associated with pollution of wastewater by Azo dyes from industrial sources have attained a significant attention^1^. It is estimated that between 10 and 15 percent of the dyes used in the textile dyeing process fail to bond with the fibers and are consequently discharged into the environment. More than 50,000 tons of organic dyes are released into the environment annually as a consequence of global dyeing processes^2^. Some surface waters have been found to contain crystal violet, a known pollutant. Treated effluents in China have been found to contain crystal violet at quantities ranging from below 0.030 g/L to over 0.049 g/L^3^. The genotoxic and mutagenic effects of these dyes have been recognized as one of the biggest areas of concern^4^. When released into water bodies through wastewater or improper disposal, these dyes can contaminate aquatic ecosystems, disrupting natural processes and harming aquatic organisms^5^. This risk is bio-accumulative that passes down through food chain^6^. In this regard, crystal violet (CV) dye is used extensively as a coloring agents in the textile industry as well as the paper industry^7^. CV is the primary component in many office supplies, including ballpoint pens, inkjet printers, and printing inks. CV dyes are used to color a wide variety of products, from fertilizers and antifreeze to detergents and leathers^8^. As a result, the CV is classified as a hazardous biological substance. The inclusion of a cationic dye in the product has been shown to cause mild eye irritation, painful photosensitization, and irreversible corneal and conjunctival damage. These effects are related to the dye’s extreme toxicity to mammalian cells^9^. In order to reduce the negative effects on eco-systems and human health; it is important to remove this dye from wastewater^10^. Wastewater can be treated via different techniques, including physical (filteration, adsorption, and coagulation-flocculation), chemical (oxidation, electrolysis, and ozonation)^11–14^. However such techniques are neither effective nor destructive when it comes to dye removal^15^.

Advanced oxidation processes (AOPs), such as the Fenton and photo-Fenton processes, which use a homogenous solution of iron (Fe^+2^) and hydrogen peroxide for the generation of hydroxyl radicals (OH^−^), might offer effective options for organic dye treatment^16–18^. On the other hand, Fenton process is limited pH reaction range (2.5–3.5); the resulted ion is regarded as a secondary pollutant^19–21^. In recent years, a wide variety of heterogeneous catalysts have been utilised to stimulate H_2_O_2_ cleavage for organic dye removal^22,23^. Recently, hydroxyapatite (HA) bio-compatible material has attracted much attention for several applications including dental implants, drug delivery, adsorbents, catalysts, and photocatalysts^24,25^. Several techniques, such as co-precipitation, sol–gel, and hydrothermal processing, can be used to synthesize HA from compounds that already include calcium and phosphorus^26–29^. HA may be generated naturally from waste by products, which has positive effects on the environment; HA has attractive properties that possess excellent characteristics as catalyst support^30^. HA has been adopted for eriochrome black-T (EBT) removal via adsorption; EBT is cancerogenic dye that carries a high level of toxicity^31^. HA nanocomposite proved to be a versatile material; HA can be adopted as adsorpent for wide range of dyes, as well as metal ions^32^. Furthermore, HA nanocomposite can find wide catalytic applications. In an attempt to enherit HA novel catalytic properties for extended applications; some structure modifications are mandatory^33^. The ionic radius of main HA constituents can offer a considerable replacement of its main ions^34^. HA structure has the ability to minimize a significant number of anionic or cationic substituents while maintaining its original crystallographic structure^35^. HA structure can be customized according to the desired application^36^. Silver is well known catalyst for hydrogen peroxide degradation^37^. The incorporation of silver into the HA matrix has the potential to deliver novel catalytic activity via superior synergistic effect with H_2_O_2_; in addition Ag ions can secure novel antimicrobial properties^38–40^. The partial replacement of calcium ion with silver has some limitations; silver ion has ionic radius of 1.28 Å compared with 0.99 Å for calcium ion^41,42^. Silver dopant induces stresses within HA lattice; the theoretical boundary for Ag^+^ doping is 20%. Moreover, the proper doping with silver ion is significantly below this limit^43,44^. It is widely believed that Ag-HA bioceramic composites may exhibit essential physicochemical features^45,46^. Furthermore, the Ag-HA nanocomposite possesses inherent antimicrobial activity, making it highly effective against microbial contamination in wastewater. Silver nanoparticles have been widely recognized for their antibacterial properties, inhibiting the growth of various pathogenic bacteria. Incorporating Ag nanoparticles into the HA matrix not only provides long-lasting antimicrobial effects but also prevents biofilm formation, reducing the risk of microbial regrowth and biofouling in wastewater treatment systems.The integration of chemisorption, photocatalysis, and antimicrobial activity in a single material, as demonstrated by the Ag-HA nanocomposite, presents a novel approach for wastewater treatment. This multifunctional bioceramic catalyst offers the advantages of pollutant adsorption, efficient degradation of organic compounds. In this study Ag-HA nanocomposite was synthesized via hydrothermal processing and the evaluation of the antimicrobial activity and photocatalytic potential of these structures for the degradation of crystal violet (CV) dye.

## Materials and methods

### Chemicals

Silver nitrate (Sigma-Aldrich, 99.99%) was employed as the precursor for silver dopant. The main precursors for HA synthesis include ammonium phosphate mono-basic (Sigma-Aldrich, 98%), and Calcium nitrate tetrahydrate (Sigma-Aldrich, 99%). Crystal violet (Sigam-Aldrich, 99%) was employed as contaminant. All chemicals were used as received without further treatment.

### Synthesis of Ag-HA nanocatalyst

HA NPs were prepared via batch hydrothermal processing according to^47^, and using 100 ml autoclave (Buchiglasuster, Switzerland). The precursors of HA synthesis include calcium nitrate solution (0.2 M) and calcium phosphate mono-basic (0.06 M). Ag-HA nanocomposite was developed through mixed nitrate solution (0.1 M silver nitrate + 0.1 M calcium nitrate) at the same synthesis conditions.

### Characterization of Ag-HA nanocatalyst

The morphology of the produced Ag-HA nanocomposite was compared to that of virgin HA using the scanning electron microscope (SEM) JEOL JEM 1010 equipment (JEOL, Tokyo, Japan), equipped with an EDAX detector (X-act, Oxford instruments) was used for elemental mapping. The study conducted TEM measurements using a JEOL JEM 1010 instrument with an acceleration voltage of 100 kV. The preparation of samples for TEM analysis involved depositing a diluted suspension of the sample onto an ultrathin carbon-coated copper grid. The study utilized a Siemens D5000 powder X-ray diffractometer to collect X-ray diffraction (XRD) patterns within the range of 2 h = 5–100, using CuKa radiation (λ = 1.54056 Å). These patterns were then compared to the crystallographic information files (CIF) obtained from the crystallographic open data base (COD). The analysis of chemical bonding in Ag-HA nanocomposite was carried out using an X-ray photoelectron spectroscopy XPS/ESCA equipment. Raman spectra of Ag-HA nancomposite was conducted via Renishaw in Via Reflex Raman spectrometer (Renishaw, Gloucestershire, UK). The FTIR spectra were examined using a JASCO FTIR 3600 spectrometer. Agilent Cary 60 UV–Vis spectrophotometer was used to study UV–Vis spectra.

### Photocatalytic degradation of crystal violet (CV) using Ag-HA nanocomposite

**Figure 1:**
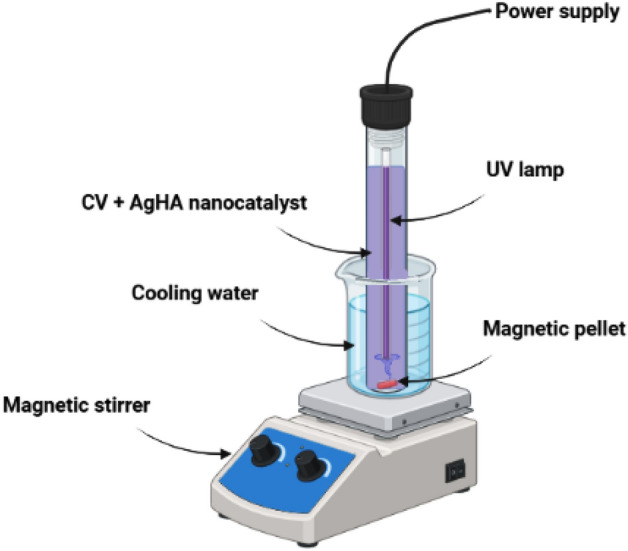
The photochemical setup used in the photocatalytic process.

Ag-HA nanocomposite (10 mg) was added to 50 ml of an aqueous CV solution with starting concentration C_0_ = 10 mg l^−1^. The mixture was stirred at 25 °C for 30 min in the dark until CV and the produced photocatalysts reached adsorption–desorption equilibrium. Hence, a quartz immersion tube with axially positioned photocatalyst and CV was irradiated with UV light from a UV lamp in the presence of Ag-HA nanocatalyst. The used UV reactor was glass cylindrical shape (100 ml) with dimensions of 3 cm diameter, 27 cm length and covered with thin film from aluminum foil. The photoreactor filled with 50 ml of contaminated solutions. The UV light irradiation source was a commercial UV-C Lamp, PHILIPS TUV 11WG11 T5, it’s a high pressure mercury lamp 11 W and having a mean wavelength 254 nm. It is totally immersed in the contaminated solutions, while the photoreactor is kept about 25 °C by a cold water bath Fig. 1 shows the photochemical setup used in the photocatalytic process. A 2.5 mm filter-equipped syringe was used to separate (1 ml) CV suspension samples at consistent irradiation intervals (10 min). The degradation rate of CV was calculated by determining the variation in CV concentration versus irradiation time using a UV–Vis spectrophotometer (Agilent Technologies Cary 60 UV–Vis) at λ max = 590 nm^48^.

### Antimicrobial activity and minimal inhibitory concentration (MIC)

The synthesized Ag-HA nanocomposite (20.0 μg/ml) were evaluated for their antimicrobial activity by agar-disc distribution method^36^, towards Bacterial strains from American Type Culture Collections (ATCC) strains, namely, Gram-negative (Escherichia coli ATCC 25,922 and (Gram-positive )Staphylococcus aureus ATCC 25,923 (bacterial strains. Conventional antibiotic discs Gentamycin (GEN); 10 μg/ml; 6.0 mm diameter), was chosen to determine the performance of the tested Ag-HA nanocomposite. The minimum inhibitory concentrations (MIC) of the tested samples which have the highest antimicrobial activity was determined by The serial dilutions method of Luria–Bertani (LB) medium^37^. For these determinations, The synthesized Ag-HA nanocomposite, and HA (beginning with concentration = 20.0 μg/ml) were applied. The medium broth act as a negative control and the medium broth inoculated with the examined microbes act as a positive control such. MIC was determined next 24 h. of incubation at 36.0 ± 1.0 °C^38^. The resultss are statistically treated by using ONE WAY ANOVA, Duncan’s multiple series, and the least significant difference (LSD) that are determined by specific software (SPSS version 15)^39^.

### Antibiofilm activity of the synthesized (AS) nanocomposite

Furthermore, a qualitative analysis concerning biofilm restraint was described as declared by Christensen et al.^49^. The biofilm’s definitive study was presented at the tube wall in the lack and closeness of the integrated Ag-HA nanocomposite was confirmed. The antibiofilm of the as-synthesized Ag-HA nanocomposite at (10.0 μg/mL) was measured against the tested microbes and was tested and correlated with the control (non-treated one). Shortly, 5 mL of the nutrient broth medium was attached inside all tubes, and the tested bacteria were inoculated, subsequent adjusted 0.5 McFarland to be 1–3.5 × 10^8^ CFU/mL. Later that, they were incubated at 37.0 ± 0.5 °C for 24 h. The media founded in control and treated tubes were dropped, combined with Phosphate Buffer Saline (PBS; pH 7.0), and ultimately preserved. Next, the bacterial cells that adhered to the tube walls were implanted with 5 mL sodium acetate (3.5%) for approximately 20 min. Finally, they were cleaned with de-ionized water. Biofilms organized inside tubes were stained with 20 mL Crystal Violet (CV; 0.15%) and washed with de-ionized water to eliminate the CV. It must be remarked that, for the semi-quantitative antibiofilm calculation, 5 mL of the absolute ethanol was injected to separate the stained bacterial biofilms^50^. UV–Vis spectrophotometer at 570.0 nm had measured the O.D. of the stained bacterial biofilms^51^. The bacterial biofilms hindrance percentage was determined by using the subsequent relation (Eq. 1)^52^:

1 $$ {\text{Biofilm inhibition}}\% \, = \left[ \left( {\text{O.D.}}\,{\text{Control sample}} - {\text{ O.D.}}\,{\text{treated sample}} \right) \, /{\text{ O.D.}}\,{\text{Control sample}} \right] \, \times \, 100 $$

## Results and discussions

### Characterization of Ag-HA nanocomposite

**Figure 2:**
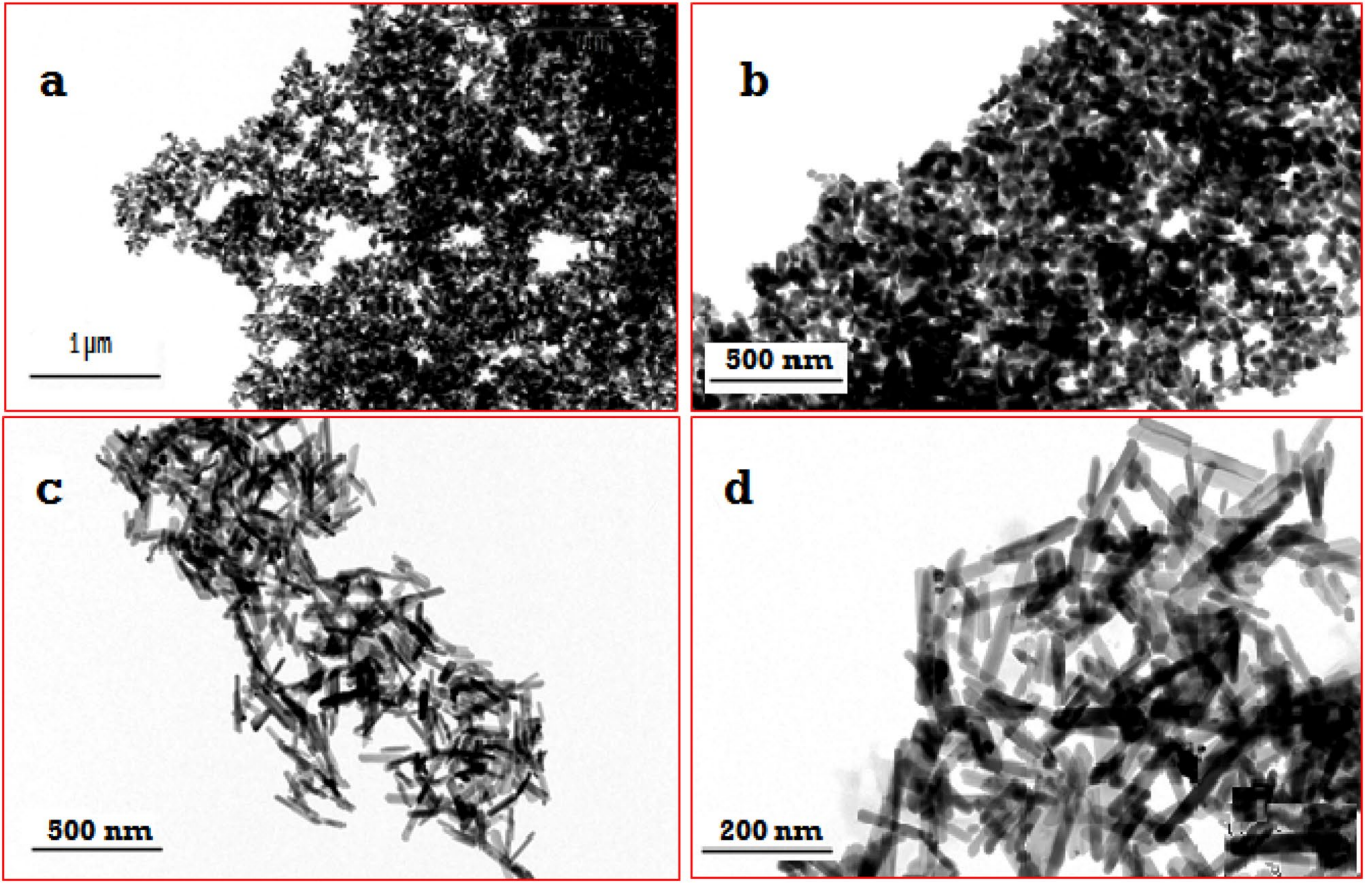
TEM micrographs of synthesized HA nanoparticles (**a**, **b**), and Ag- HA nanorods (**c**, **d**).

Using TEM, the morphology of synthesized HA revealed high-quality monodispersed particles of 20 nm (Fig. 2a,b). Integrating Ag ions into the HA structure resulted in a profound alteration of the HA structure. Figure 2c,d demonstrates that the Ag-HA nanocomposite generated monodispersed nanorods measuring 60 nm in length and 6 nm in diameter.

**Figure 3:**
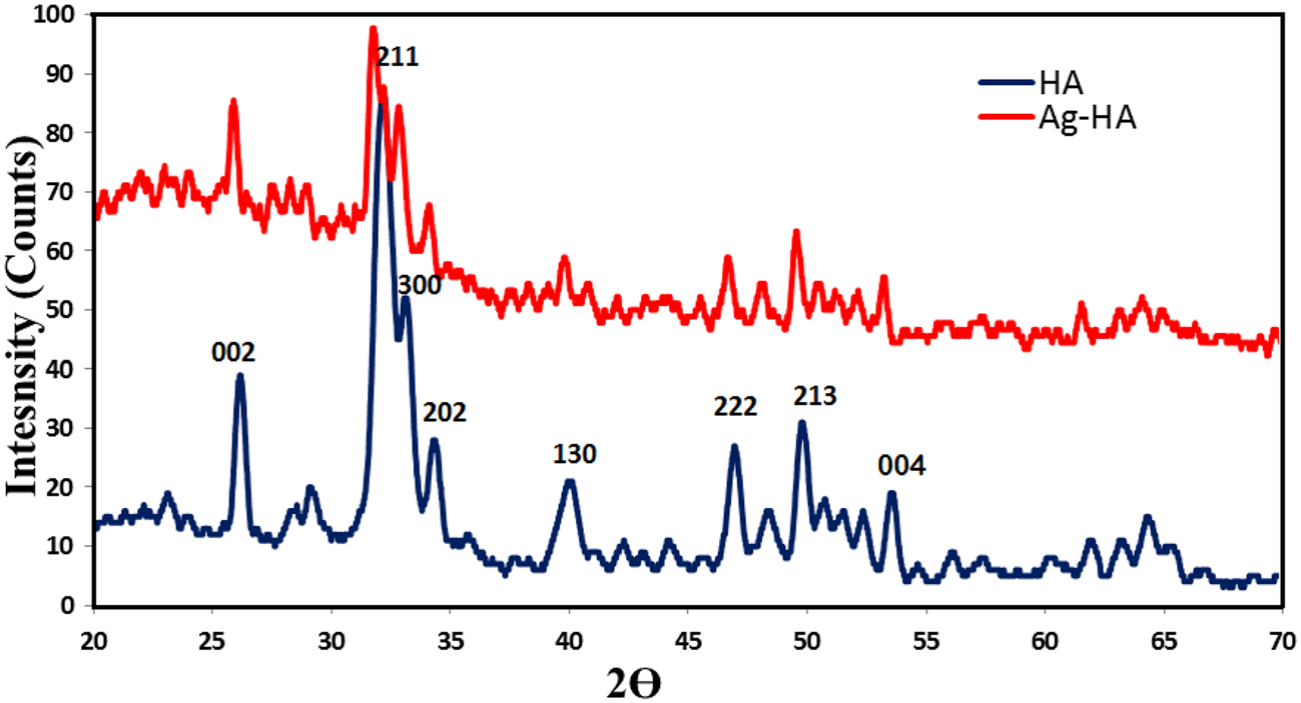
XRD diffractogram of Ag-HA nanocomposite.

The crystalline structure of dry particles was investigated with XRD. Ag-HA nanocomposite exhibited a highly crystalline structure; the crystalline structure of HA was not altered. In addition, Ag-HA exhibited nine characteristic peaks similar to those of HA. In accordance with the ICCD card number 00-024-00 (Fig. 3), these distinctive peaks were discovered. XRD diffractogram revealed highly crystalline HA particles.

The uniform distribution of silver ions in the HA structure was measured with an EDAX detector and elemental mapping of an Ag-HA nanocomposite (Fig. S1).

**Figure 4:**
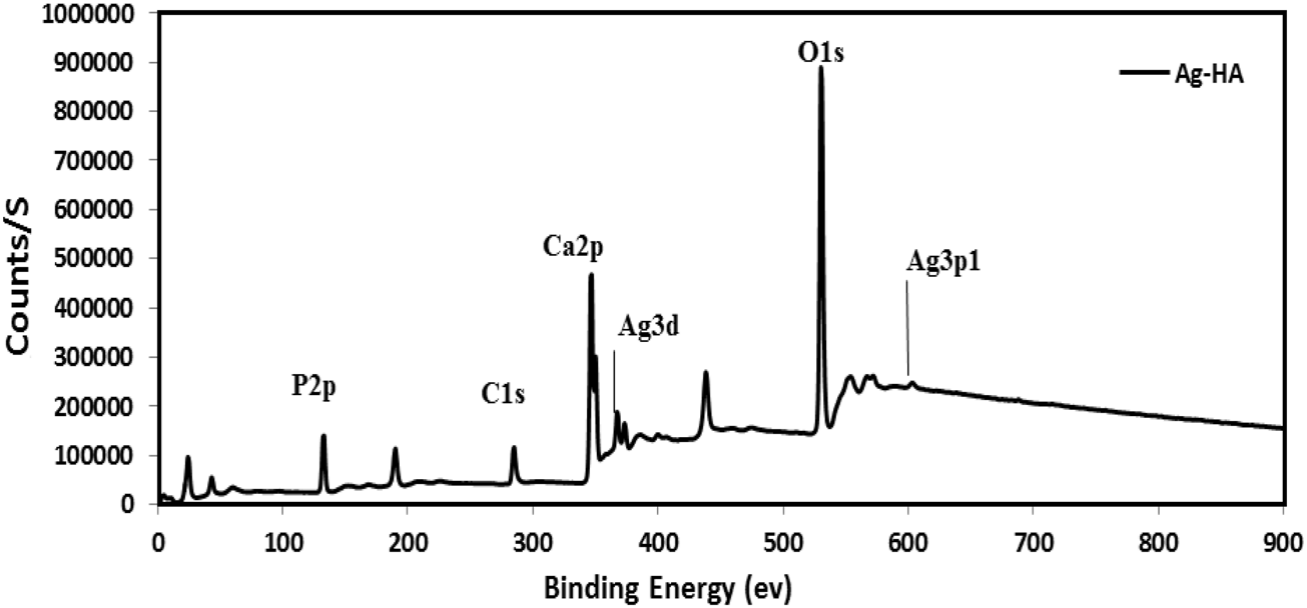
Spectra of Ag-HA nanocomposite via XPS.

The successful generation of Ag-HA nanocomposites was validated by the uniform distribution of Ag^+^ in the HA matrix. Using XPS spectroscopy, the elemental composition of Ag-HA was quantified. The precise elemental binding energies of C_1_S at 284.8 eV were determined. Figure 4 depicts the Ag-HA nanocomposite XPS survey spectra.

**Figure 5:**
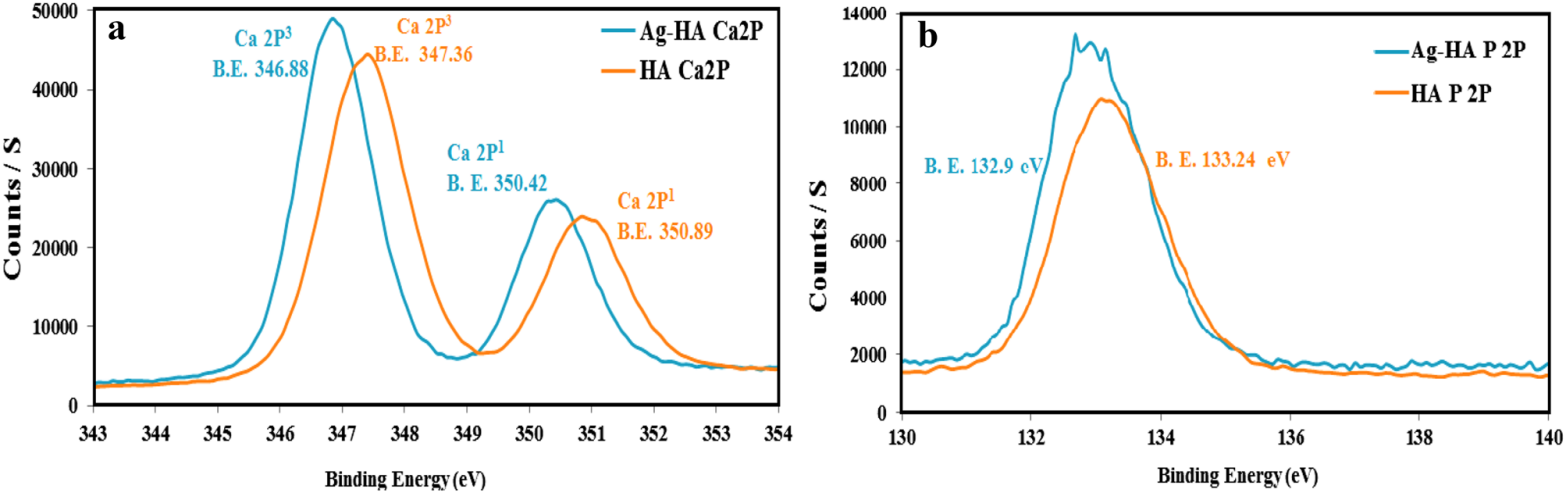
Impact of silver ion on binding energies of Ca2P (**a**), and P2P (**b**).

The atomic percentage of silver ions was restricted to 1%. Silver has a larger ionic radius (1.28 Å)) than calcium ion (0.99 Å); as a result, the silver content in Ag-HA can be limited^41^. The theoretical upper limit for Ag^+^ dopant is 20%. However, the silver ion content is significantly lower^43,44^. The ca/P ratio of the Ag-HA nanocatalyst was 1.238 compared to the theoretical value of 1.405. Due to the partial substitution of Ca^2+^ with Ag^+^ ion, the Ca/P ratio has decreased. The presence of silver dopant had a significant effect on the calcium ion binding energies (Fig. 5a). Ca 2P^3^ exhibited a binding energy of 347.36 eV for pure HA and 346.88 eV for Ag-HA nanocomposite. (Fig. 5b) Silver dopant decreased the binding energy of phosphorous ions.

**Figure 6:**
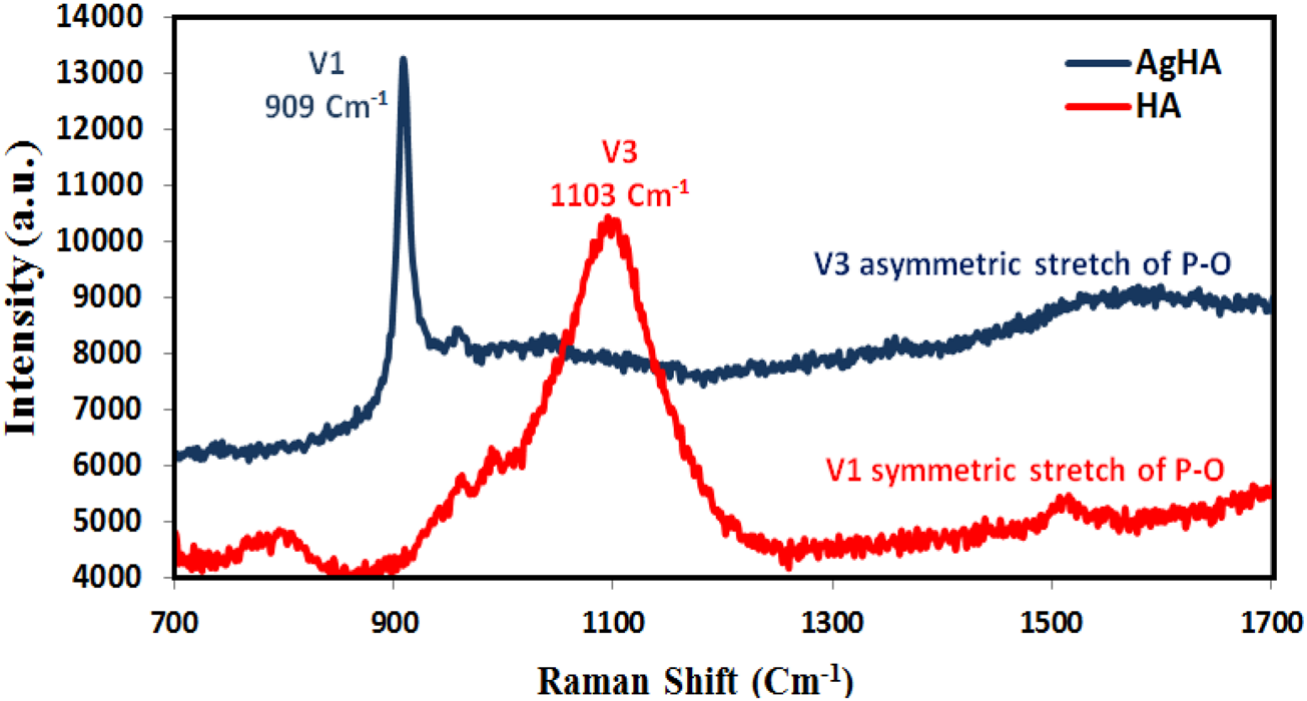
Raman spectra of Ha and Ag-HA nanocomposite.

Silver dopant demonstrated not only a significant impact on HA morphology; but also decrease the binding energy (B.E.) of its main constituents. It can be concluded that the valence electrons of Ca and P ions are less tightly bound to their respective atoms; as a result, they are easily excitable and readily participate in chemical reactions^24^. The enhanced photocatalytic activity could therefore be achieved. The optical properties of Ag-HA nanocomposite were investigated further using a UV–Vis spectrometer. Compared to pure HA, Ag-HA nanocomposite exhibited high light absorption over the UV–Visible band 200–800 nm (Fig. S2a). From photolumensence studies (Fig. S2b) we can conclude that, the Ag-HA nanocomposite has low absorbance in the visible regions and high absorbance in the ultraviolet region^50^. the UV absorption band is observed in the region 210–490 nm, which originates primarily from the absorption and scattering of light by the Ag-HA nanocomposite.The decrease in the B.E. of valence electrons may account for the enhanced Ag-HA absorbance. Using Raman spectroscopy, the chemical structure of Ag-HA nanocomposite was investigated. Raman spectra can provide high sensitivity to the secondary phase. The P-O asymmetries can be attributed to the significant absorption observed at 1103 cm^−1^ in pure HA; however, this peak was considerably diminished in the Ag-HA sample. The symmetric P-O stretch was shifted to a lower frequency at 909 cm^−1^ (Fig. 6). This change in the P-O stretch may be attributable to the presence of the Ag dopant and its effect on the B. E. of valance electrons.

Figure S3 shows that FTIR spectroscopy was used to compare the functional groups of Ag-HA to those of pure HA nanoparticles. According to FTIR spectra, Ag-HA nanocomposite exhibited a hydroxyapatite signature. In addition, Ag-HA exhibited intensive absorption at 3430 cm^−1^; silver doping could reduce the B.E. of oxygen valence electrons on the surface. This characteristic may facilitate the formation of hydrogen bonds and enhance infrared absorption^53,54^. In an apatitic environment, the following bands were observed: 559 cm^−1^, 757 cm^−1^, 603 cm^−1^, 960 cm^−1^, and 1000–1100 cm^−1^, for the PO_4_^3−^ groups^55^ and at 875 cm^−1^ for the HPO_4_^2−^ ions^56^.

### Impact of silver dopant on photocatalytic efficiency

**Figure 7:**
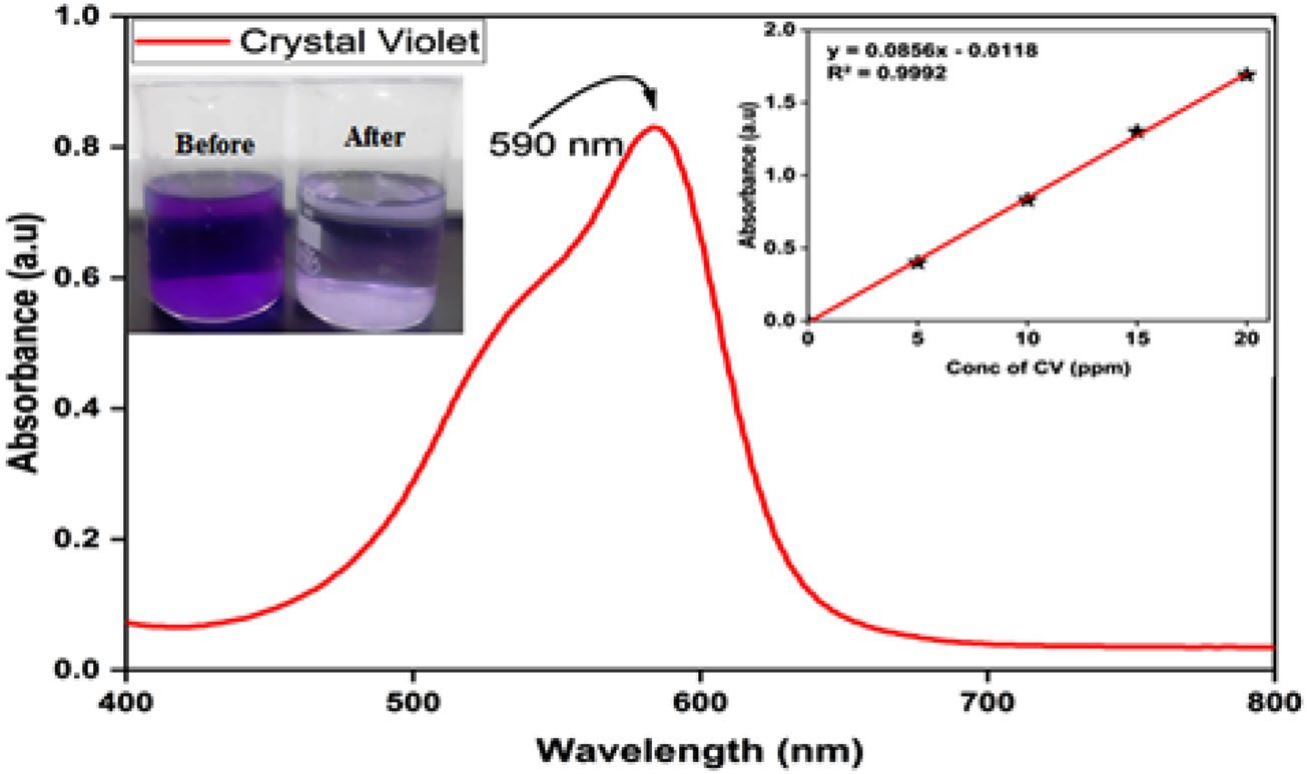
UV–Vis. spectrum of CV dye and calibration curve at different CV concentrations.

It is generally accepted that most dyes resist biodegradation and direct photolysis. However, many nitrogen-containing dyes, such as CV, endure natural reductive anaerobic degradation to produce potentially carcinogenic aromatic amines^57^. To measure the photocatalytic activity of Ag-HA nanocomposites, CV was used as a model contaminant. At λ max = 590 nm, the CV removal was determined spectrophotometrically (Fig. 7)^58^.

HA can be employed for decontamination via adsorption mechanism due to hydroxyl function groups. The impact of Ag dopant on HA removal efficiency via chemisorption was evaluated under dark conditions for 4 h. Virgin HA demonstrated removal efficiency of 7.9% compared with 11.8% for Ag-HA nanocomposite (Fig. S4). Silver dopant could decrease the binding energy (B.E.) of valence electrons of surface hydroxyl groups. This could intensify the negative charge on the oxygen; offering high chemosoprtion of positively charged CV dye^59^.

### Photocatalytic activity of Ag-HA nanocomposite

**Figure 8:**
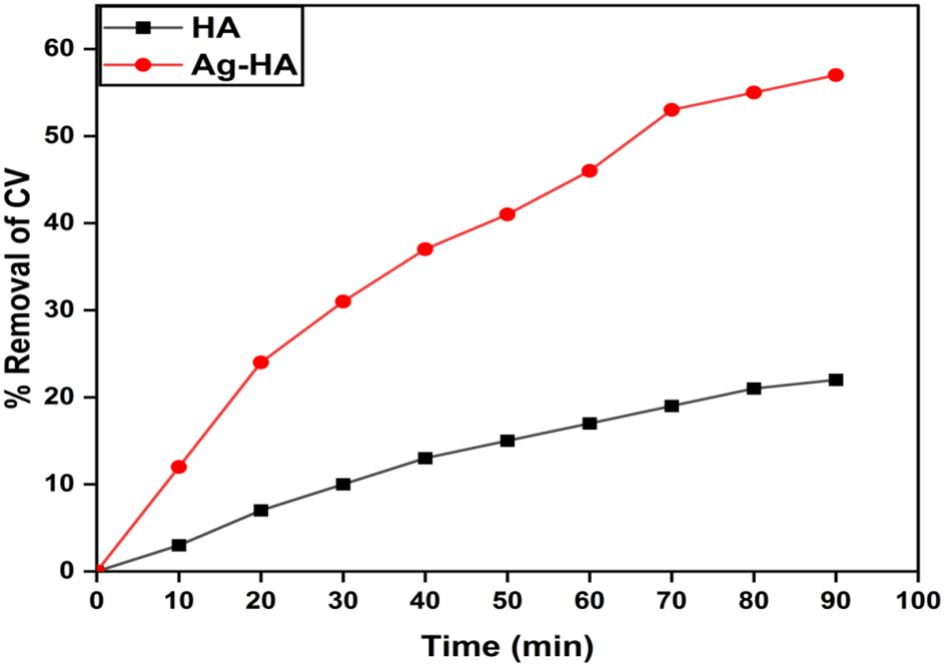
Percent of CV removal by Ag-HA nanocomposite to virgin HA as a function of time under UV irradiation.

Ag-HA nanocomposite demonstrated removal efficiency of 57% compared with 22% for virgin HA, after 90 min. under UV irradiation (Fig. 8). Comparative removal efficiency between dark and under UV irradiation demonstrated that most Ag-HA nanocomposite removal could be correlated to superior photocatalytic activity of Ag-HA nanocomposite.

The superior photocatalytic activity of Ag-HA nanocomposite could be ascribed to silver dopant that could offer novel characteristics such as enhanced electron–hole generation, extended light absorption into the UV–Vis. Range, and enhanced interfacial charge transfer efficiency^60^. Additionally, Increasing light intensity can generally enhance the rate of photo-catalytic reactions. The higher the light intensity, the greater the number of photons available to be absorbed by the catalyst. This results in more excited electrons and a higher probability of successful catalyst-substrate interactions, leading to increased reaction rates. However, there is a limit to this effect, as excessive light intensity may cause thermal effects or even lead to catalyst degradation^61^.

The wavelength of the UV light used in a photo-catalytic reaction can also influence the reaction rate and selectivity. Different catalyst materials have specific absorption bands, and their efficiency in utilizing light energy varies with the wavelength. By changing the UV light wavelength, you can tune the absorption characteristics of the catalyst and optimize its performance^62^.

#### Effect of pH on CV removal

**Figure 9:**
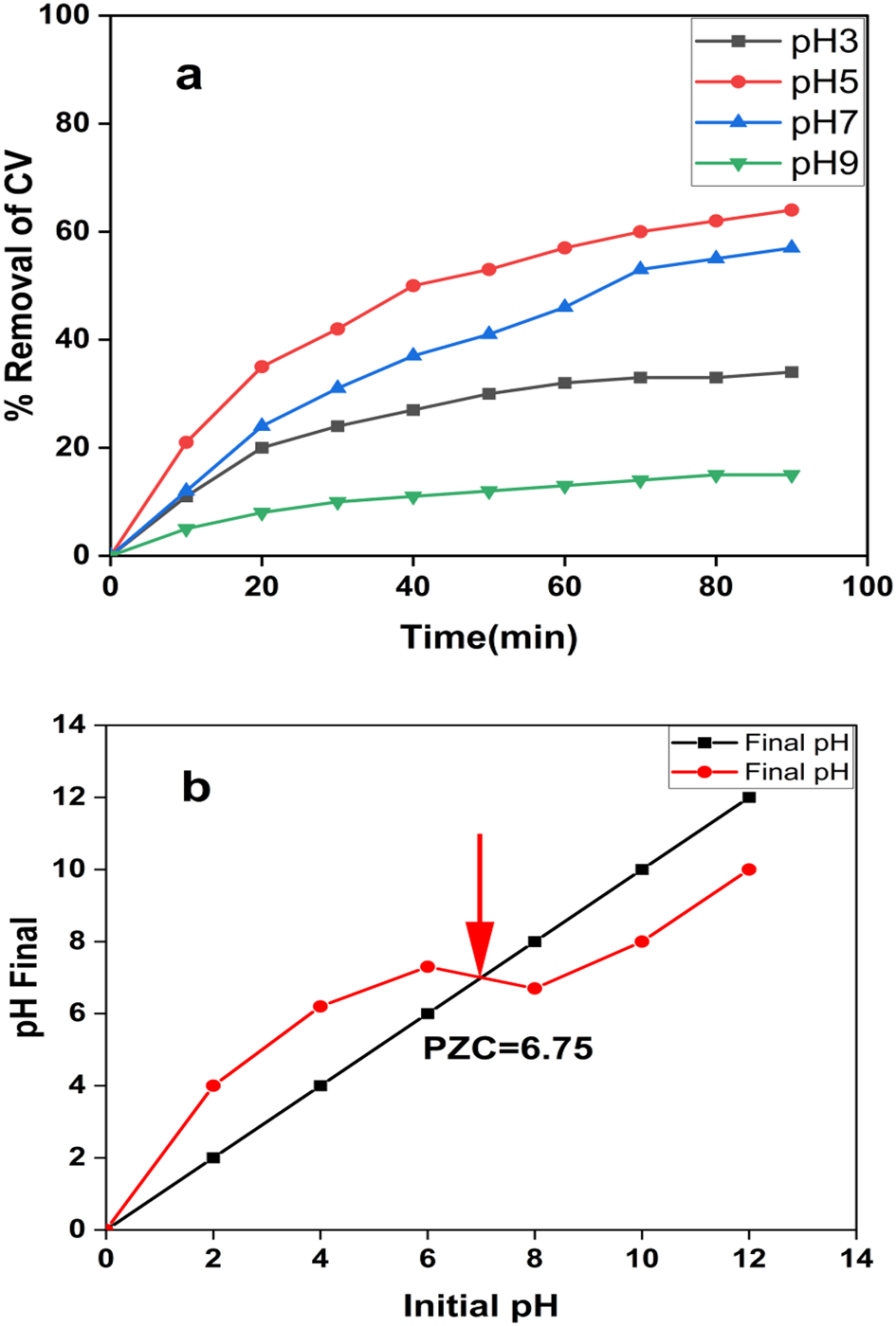
(**a**) The variation of CV removal (%) with time at different solution pH (3.0, 5.0, 7.0 and 9.0) (10 mg g of Ag-HA in 50 ml of 10 ppm CV at 25 °C), and (**b**) Point of zero charges (PZC) of Ag-HA at different pH values.

The significant impact of initial pH values on CV removal was investigated for 90 min under specified experimental conditions (10 mg of the prepared nanocomposite, 50 ml of 10 ppm CV solution, Temp. = 25 °C). The variation of CV removal (%) with time at different solution pH values (3.0–9.0) is demonstrated in Fig. 9a. The maximum CV removal (62%) in equilibrium was accomplished at pH 9.0. To determine the point of zero charges (PZC) of the Ag-HA NPs, 0.01 g (Ag-HA NPs) was added to 50 mL (0.01 M NaCl solution). The pH levels of the solution were adjusted to 2, 4, 6, 8, 10, and 12 using HCl or NaOH . The samples were mixed for 48 h at a speed of 200 rpm. After the separation of (Ag-HA NPs), then the pH levels of the solutions were determined. A graph that demonstrates the relationship between the final pH and the initial pH was obtained in order to calculate the pH of the PZC value. PZC was discovered to have existed at a pH value of 6.75, which is demonstrated in Fig. 9-b.

PZC occurred in regions where there was no substantial difference between the final and initial pH values. When pH is less than or greater than PZC, the surface charge of the Ag-HA nanocomposite will be positively charged, and when pH is greater than or equal to PZC, the charge will be negatively charged. Also, when the pH of the solution is equal to the pH of the PZC, the photocatalyst surface charge is neutral, and the electrostatic force between the photocatalyst surface and ions (CV ions) is negligible. This occurs when the solution is at a pH that is identical to the pH of the PZC^63^. Based on PZC value, the optimum photocatalytic efficiency of Ag-HA nanocomposite was reported at pH 9 (Fig. 9a); this could be ascribed to the negatively charged Ag-HA surface. Negative sites could attract the positive charge sites of CV. This feature could offer a high capability to act as an electron donor and to initiate photocatalytic degradation of CV contaminants. This electrostatic attraction would improve CV photocatalytic degradation. On the other hand, the removal of CV began to decrease at pH = 5.0. The surface of Ag-HA nanocomposite becomes positive at this pH value. The repulsive force would be evolved between CV positive charge and Ag-HA nanocomposite at pH < 6.55. This could withstand low removal efficiency at a low pH value.

#### Effect of CV initial concentration and nanocomposite dose on removal efficiency

**Figure 10:**
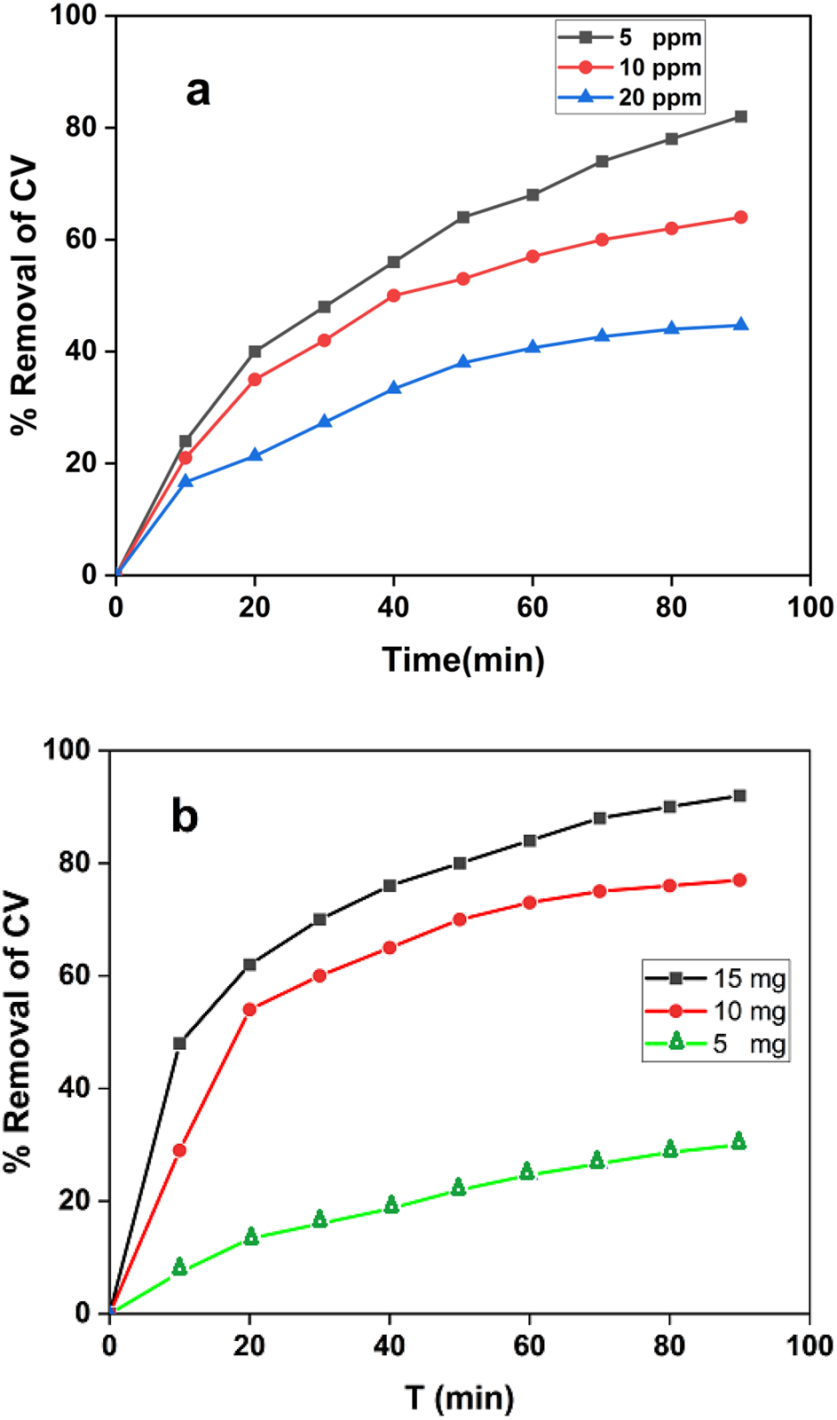
(**a**) The variation of percent removal as a function of contact time at different initial CV concentrations (5, 10, and 20 ppm) at pH 9 and 10.0 mg Ag-HA, and (**b**) Effect of the photocatalyst dose on the removal efficiency of CV (50 ml CV solution (10 ppm), Temp. = 25 °C and pH 9).

Initial CV concentration plays an important role in removal efficiency. The impact of CV ionic strength was investigated by varying initial CV concentration while maintaining other reaction conditions with no change. Figure 10a represents the variation of removal % as a function of contact time at different initial CV concentrations (5.0, 10.0 and 20.0 ppm). The results demonstrated that degradation efficiency is inversely proportional to the concentration of CV, which can be effectively removed in the presence of the prepared nanocomposite under UV light irradiation even at high initial concentrations.

The influence of Ag-HA nanocomposite dose on CV removal efficiency under UV-light was investigated. The removal efficiency was investigated as a function of time for different initial Ag-HA nanocomposite masses for a fixed concentration of CV (10 ppm) (Fig. 10b). The obtained results demonstrated an increase in the removal efficiency with the increase in the photocatalyst dose from (5 to 15 mg). The observed increase in removal efficiency with photocatalyst amount, could be attributed to the increase in the available active area or active sites of the photocatalyst to volume ratio of CV solution^64^.

#### Kinetic studies

Figure S5 shows the pseudo-first-order and pseudo-second-order kinetic plots for the CV removal from solution with different initial CV concentration using Ag-HA at 25 °C. From the correlation coefficient R^2^, it can be seen that the removal kinetics data for Ag-HA fit better with the pseudo-first order kinetic model.

The degradation rate of CV can be calculated via Eq. (2):

2 $$ - \ln \, \,{\text{C}}_{{\text{t}}} /{\text{C}}_{{\text{O}}} = \, - {\text{ Kt}} $$

where C_t_ and C_o_ are the remaining and the initial concentrations of CV respectively, t is the removal time, and k is the removal rate constant. The relation of—ln (C_t_/C_o_) vs. t. is represented in Fig. S5. On the basis of the results, it was determined that the kinetics of the removal reaction followed pseudo-first-order rate laws. Furthermore Fig. S5 revealed that an increase in the CV initial concentration results in a drop in the apparent pseudo-first-order rate constants. This was proved by the correlation between the two variables. This dependence on reaction rate constants on CV concentration was found to be in good accordance literature^65,66^.

### Synergistic catalytic effect of Ag-HA with H_2_O_2_

**Figure 11:**
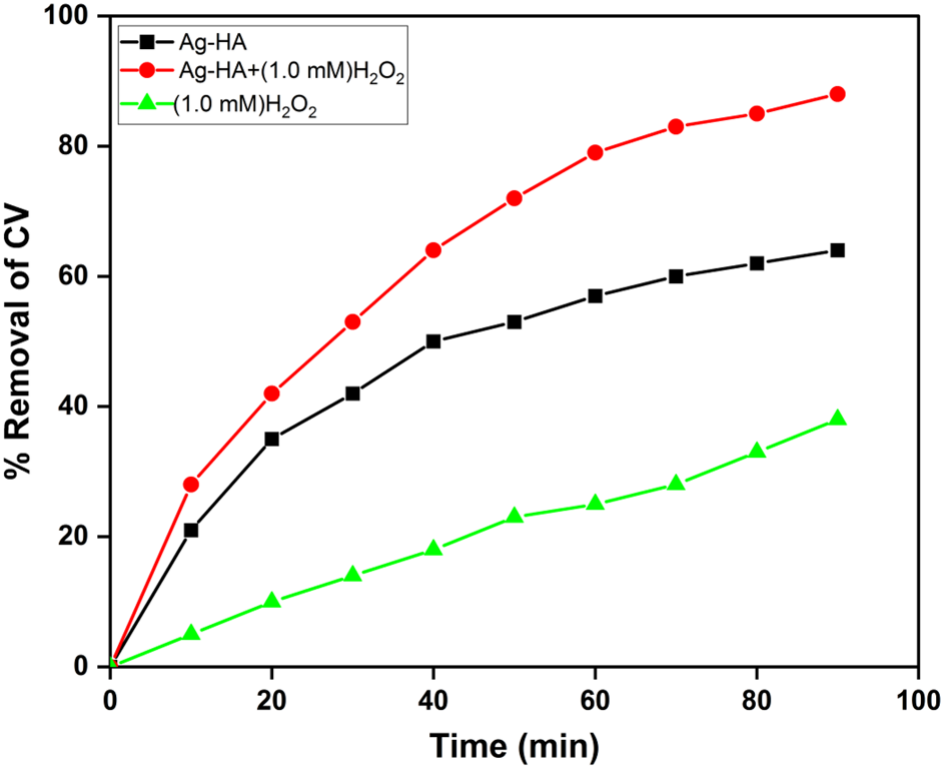
Removal percentage of Crystal violet in the presence of (1.0 mM) hydrogen peroxide under UV light irradiation and 10 mg catalyst, 50 mL of 10 ppm dye concentration.

Comparison of removal efficiency in the dark and light irradiation conditions demonstrated that most of the CV removal could be due to photocatalytic degradation. Ag-HA nanocomposite demonstrated removal efficiency of 57% under UV irradiation in 90 min. Silver dopant could facilitate charge separation and the absorption of incident light. Silver itself is the universal catalyst for H_2_O_2_ decomposition. Therefore enhancement of the photocatalytic activity of Ag-HA nanocomposite in the presence of H_2_O_2_ is expected. The superior removal performance of Ag-HA nanocomposite in the presence of H_2_O_2_ is demonstrated in Fig. 11.

Hydrogen peroxide alone did not show noticeable contaminant degradation. Silver dopant could initiate decomposition of H_2_O_2_ to generate ȮH radicals; these radicals could contribute to the CV photodegradation. Furthermore, silver dopant could increase the photocatalysis rate via enhancement of electron–hole generation.

### Mechanism of photocatalytic activity of Ag-HA nanocomposite

Silver dopant could induce novel photocatalytic mechanism within Ag-HA nanocomposite under UV irradiation via electron transfer from the highest occupied molecular orbital (HOMO) of O and P atoms to the lowest unoccupied molecular orbital (LUMO) of Ag^67,68^. The HOMO orbitals are absolutely necessary, for an electron to be brought back to its stable condition. The generation of the (ȮH) radical therefore requires the removal of one electron from the water molecule. The active (ȮH) radicals function as a powerful oxidising agent to cleave CV molecules and create the final oxidation products. This is accomplished by cleaving the CV molecules. It is important to highlight that there is a synergetic effect that results from the interaction of H_2_O_2_ and Ag-HA nanocomposite, which forms (ȮH) and HO_2_^−^ radicals. This impact should be considered significant^67^. In the following equations, we see a demonstration of two critical phases that correspond to the cleavage of H_2_O_2_ by silver dopant:

3 $$ {\text{Ag}} - {\text{HA }}\,{\text{NPs }} + {\text{ H}}_{2} {\text{O}}_{2} \to^{ + } {\text{Ag}} - {\text{HANPs }} + {\text{ HO}}_{{2^{ \cdot } }} + {\text{ H}}^{ + } $$

4 $$^{ + } {\text{Ag - HA }}\,{\text{NPs }} + {\text{ H}}_{{2}} {\text{O}}_{{2}} \to {\text{Ag - HA}}\,{\text{ NPs }} + {}^{ \cdot }{\text{OH }} + {\text{ OH}}^{ - } $$

5 $$ \left[ {3} \right] \, + \, \left[ {4} \right]: \, + {\text{ 2 H}}_{{2}} {\text{O}}_{{2}} \mathop{\longrightarrow}\limits^{{{\text{Ag - HA}}\,{\text{nanocatalyst}}}}{}^{ \cdot }{\text{OH}} + {\text{ HO}}_{{{2}^{ \cdot } }} + {\text{ H}}_{{2}} {\text{O}} $$

**Figure 12:**
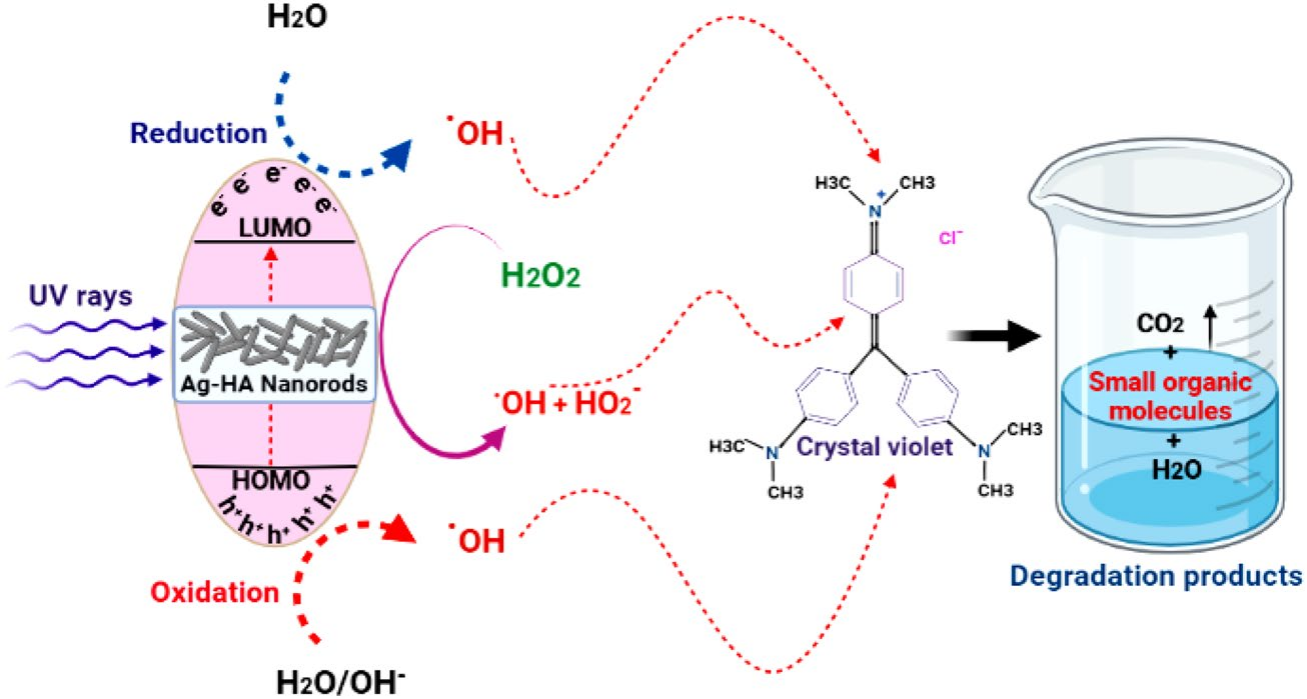
Proposed mechanism of photocatalytic degradation of crystal voilet by the prepared Ag-doped hydroxyapatite (Ag-HA) nanorods in the presence of H_2_O_2_.

In the case of the photodegradation of CV, in addition to the photocatalytic activity of the Ag-HA nanocatalyst, the synergistic effect that occurs between Ag-HA and H_2_O_2_ significantly accelerates the entire photodegradation process. This is accomplished by the production of more active (ȮH) radicals. Figure 12 is a representation of the suggested photocatalytic mechanism of Ag-HA nanocomposite when it is present in an environment containing H_2_O_2_.

### Antimicrobial activity of the synthesized (HA) and (Ag-HA) nanocomposite

**Figure 13:**
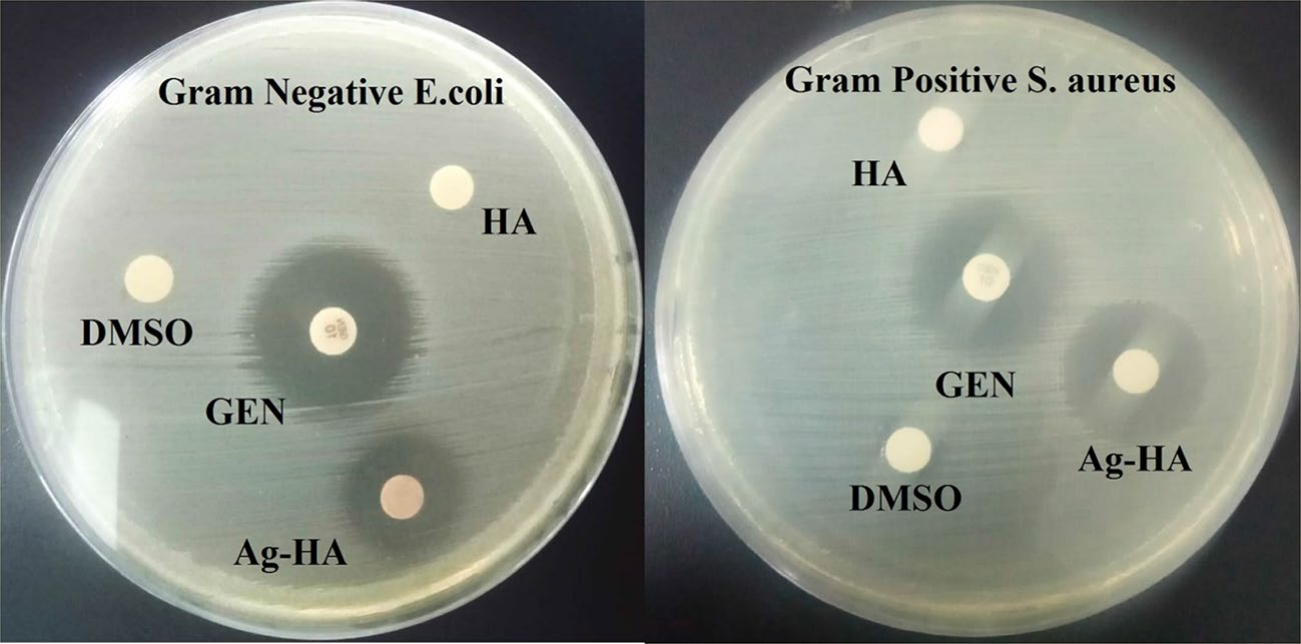
Antibacterial activity of prepared sample against (**a**) gram positive (*S.aureus*) bacteria, and (**b**) gram negative (*E. coli*) bacteria.

Silver dopant could inherit HA novel anti-microbial activity. The in-vitro ZOI result verified that the synthesized Ag-HA nanocomposite exhibited enhanced antibacterial activity against *S. aureus* (18 mm ZOI), and *E. coli* (14 mm ZOI) as displayed in Fig. 13, and listed in Table 1. It is worth considering that the antibacterial potency of (Ag-HA) nanocomposite was significantly more powerful than (HA) NPs which suggested the synergistic effect of silver dopant. Bhattacharjee *etal.*, verified that no significant zone of inhibition was found for undoped hydroxy apatite but incorporation of Zn and Co leads to antibacterial efficacy against *E coli*^69^. Silver nanoparticles can release silver ions when exposed to an aqueous environment. These ions can interact with various biomolecules and potentially exhibit antimicrobial activity^70^. The presence of electronic effects, which come from changes in the local electronic structure on the surfaces of smaller-sized particles, is linked to the bactericidal action of Ag nanoparticles. These actions are thought to contribute to an increase in the reactivity of Ag nanoparticle surfaces^68^. It was reported that Ag ion strongly interacts with the thiol group of vital enzymes and inactivates them^71^. It is important to assume that; the incorporated (Ag-HA) nanocomposite was active against Gram-positive bacteria more than Gram-negative, this is maybe due to that the cell walls constituents in Gram-negative bacteria contain principally little layers of lipopolysaccharide, lipid, and peptidoglycan. On the other hand, the cell walls of Gram-positive incorporate very solid peptidoglycan forms^50,72^.

**Table 1:** Antimicrobial activities of (HA) NPs and (Ag-HA) nanocomposite against gram-positive and gram-negative bacteria measured as ZOI (mm) and MIC (µg/ml).

Pathogenic bacteria	ZOI of HA (10.0 µg/ml) (mm)	ZOI of Ag-HA (10.0 µg/ml) (mm)	ZOI of GEN (10.0 µg/ml) (mm)	MIC of Ag-HA (µg/ml)	% Biofilm inhibition by Ag-HA (10 μg/ml)
*S. aureus*	− ve	18.0 ± 0.21	22.0 ± 17	0.625	91.1
*E. coli*	− ve	14.0 ± 14	18.0 ± 35	1.250	73.2

The MIC results of (Ag-HA) nanocomposite against *S. aureus* and *E. coli* were 0.625 and 1.250 μg/ml respectively as mentioned in Table 1. The synthesis of exo-polysaccharides can be used to determine whether or not pathogenic microorganisms have begun to produce biofilm^73^. The tube method is used to qualitatively define the antibiofilm behavior of Ag-HA nanocomposite toward various pathogenic bacteria and unicellular fungi^74^. A UV–Vis. spectrophotometer is examined for the semi-quantitative measurement of the inhibition %. The optical density (O.D.) was measured at 590 nm following terminating CV-stained biofilms, which is recognized as a power of their production^75,76^. According to Table 1, the maximum percentage of biofilm inhibition achieved for *S. aureus* is 91.1%, while the highest percentage achieved for *E. coli* is 73.2%. It is essential to remember that Ag-HA nanocomposite is capable of controlling the extension of biofilm at its adhesion strength, which is the first step in the process of eliminating microbes^77^.

### Mechanism of antimicrobial activity of the synthesized Ag-HA nanocomposite

**Figure 14:**
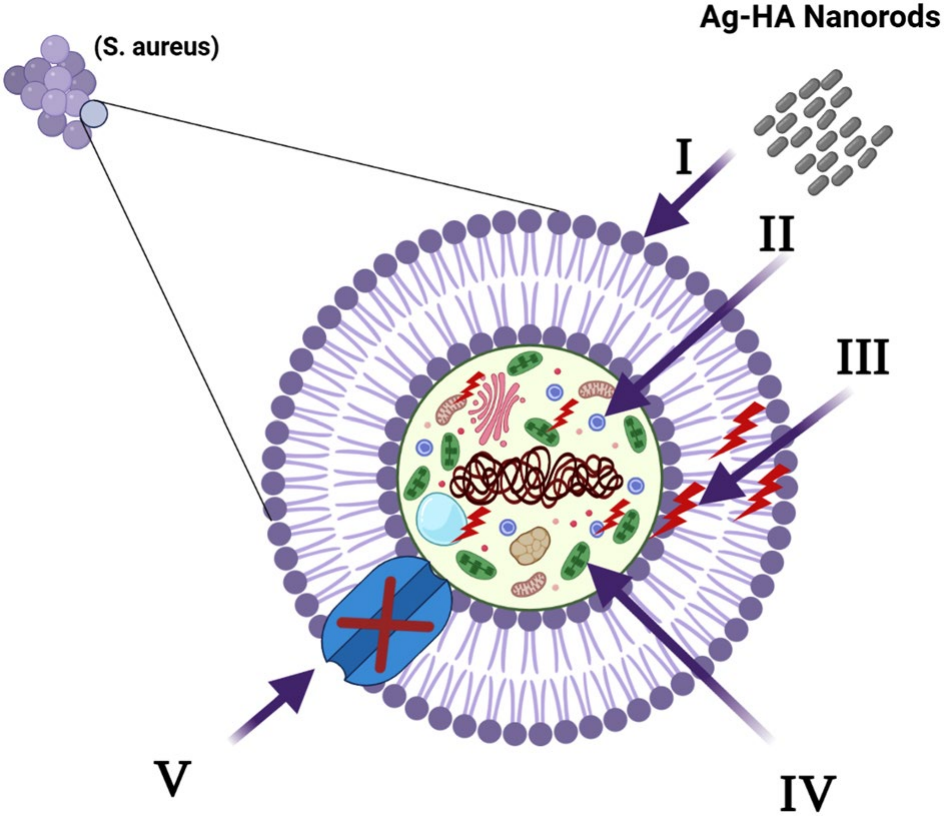
Schematic representation of the four main pathways underlying the antibacterial potential of Ag-HA nanocomposites: (I) the Ag-HA nanocomposite adhere to and wrap the microbial cell surface, resulting in the release of silver ions, causing membrane damage and altered transport activity. (II) Ag-HA nanocomposite penetrate the microbial cells and interact with cellular organelles and biomolecules (such as plasmid DNA, ribosomes, chromosomal DNA, and mesosomes), affecting the respective cellular machinery. (III) Ag-HA nanocomposite creates and increases ROS, leading to cell damage. (IV) Ag-HA nanocomposite modulate the cellular signal system and causing cell death. (V) Finally, Ag-HA nanocomposite blocks the ion transport from and to the microbial cells.

The proposed antibacterial mechanism is depicted schematically in Fig. 14. First, the Ag-HA nanocomposites wrap around and adhere to the microbial cells’ outer surface, rupturing their membranes and altering their transport capacity^56^. Then, all internal components, including plasmid, DNA, and other crucial organelles, are divided by the dispersion of the Ag-HA nanoparticles inside the microbial cell. Ultimately, Cellular toxicity ultimately results from the oxidative stress brought on by the production of ROS. Finally, nano-composite prevent the transfer of ions to and from microbial cells^57^.

## Conclusion

High quality silver dopped HA nanorods of 60 nm lenth was developed. Silver dopant was reported to intensify surface negative charge and enhanced the chemosorption of positively charged CV dye. It was confirmed that the Ag-HA NPs showed the highest UV-photocatalytic activities for CV removel (64% removal efficiency after 90 min). Removal efficiency reached 88% through the synergistic effect of silver dopant with 1.0 mM H_2_O_2_. Additionally photocatalytic wastewater treatment can offer several potential advantages:

- Photocatalysis can effectively degrade various organic pollutants, including complex and recalcitrant compounds, potentially achieving higher levels of treatment compared to conventional processes.
- Photocatalytic systems typically require less energy especially if natural sunlight or low-energy UV sources are used.
- Photocatalytic reactors can be designed with smaller footprints, making them suitable for decentralized applications or retrofitting existing treatment plants.
- Photocatalysis can facilitate the breakdown of organic compounds into simpler forms, potentially enabling resource recovery or further treatment of the by-products.

However, it’s important to note that the cost-effectiveness of photocatalytic wastewater treatment can vary depending on factors such as the specific contaminants present, the scale of the treatment system, availability of suitable catalysts, maintenance requirements, and local energy costs.

On the other hand, Silver dopant inherited HA novel anti-bactrial activity. Ag-HA nanocomposite was active against Gram-positive (*S. aureus*) further than Gram-negative (*E. coli*) bacteria (22.0 and 18.0 mm ZOI, respectively). After the addition of 10.0 g/mL Ag-HA nanocomposite, the percentage of biofilm inhibition shows that the maximum percentage for *S. aureus* is 91.1%, while the inhibition percentage for *E. coli* is 73.2%. Our work provides a revolutionary, nanomaterial-based, and cost-effective solution for wastewater treatment to assist in solving global water shortage issues. Other applications for Ag-HA include periodontal treatment, maxillofacial surgery, alveolar ridge augmentation, and otolaryngology need furture studies in the future work.

### Supplementary Information

Supplementary Figures.

## Data Availability

The datasets used and/or analysed during the current study available from the corresponding author on reasonable request.
