# Supplementary material for: Enhanced photocatalytic and antibacterial activities of novel Ag-HA bioceramic nanocatalyst for waste-water treatment
Source: Sci Rep. 2023 Aug 24;13:13819. doi: 10.1038/s41598-023-40970-4 (PMC10449880; doi:10.1038/s41598-023-40970-4)
Supplement: Supplementary file 1 — Supplementary Figures. [file 41598_2023_40970_MOESM1_ESM.docx]

**Enhanced Photocatalytic and Antibacterial Activities of Novel Ag-HA Bioceramic Nanocatalyst for Waste-water Treatment**

**Sherif Elbasuney^a*^, Mohamed A. Elsayed^b^, Ahmed M. El-Khawaga^c*^, Miguel A. Correa-Duarte^d^**

^a^ Head of Nanotechnology Research Center, Military Technical College, Egyptian Armed Forces, Cairo, Egypt.

^b^ Head of School of Chemical engineering, Military Technical College, Cairo, Egypt.

^c^ Department of Basic Medical Sciences, Faculty of Medicine, Galala University, New Galala City, Suez, Egypt**.**

^d^ Department of Physical Chemistry, Biomedical Research Center (CINBIO), and Institute of Biomedical Research of Ourense-Pontevedra-Vigo (IBI), Universidad de Vigo, 36310 Vigo, Spain.

*Corresponding Authors E-mail**:**

**1-** Sherif Elbasuney: [sherif_basuney2000@yahoo.com](mailto:sherif_basuney2000@yahoo.com), [s.elbasuney@mtc.edu.eg](mailto:s.elbasuney@mtc.edu.eg)

**2-**Ahmed M. El-Khawaga: Ahmed.Elkhawaga@gu.edu.eg, [Ahmedelkhwaga15@gmail.com](mailto:Ahmedelkhwaga15@gmail.com)


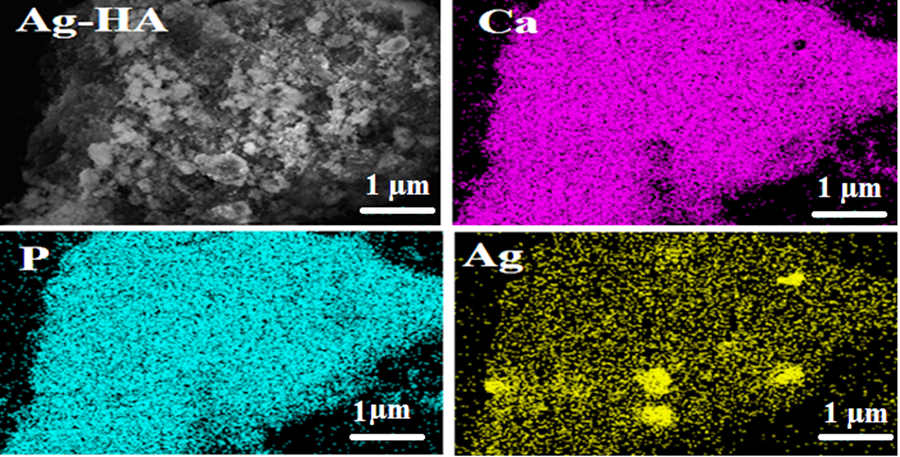


**Fig. S1: Elemental mapping of silver ion within HA matrix via EDAX detector.**

**
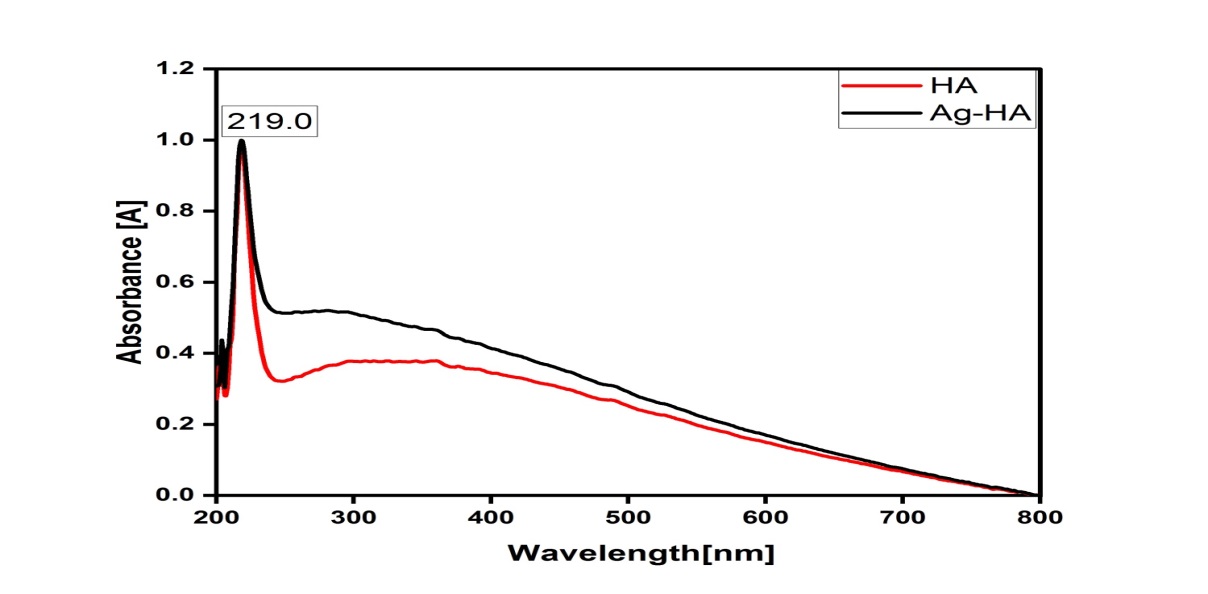
**
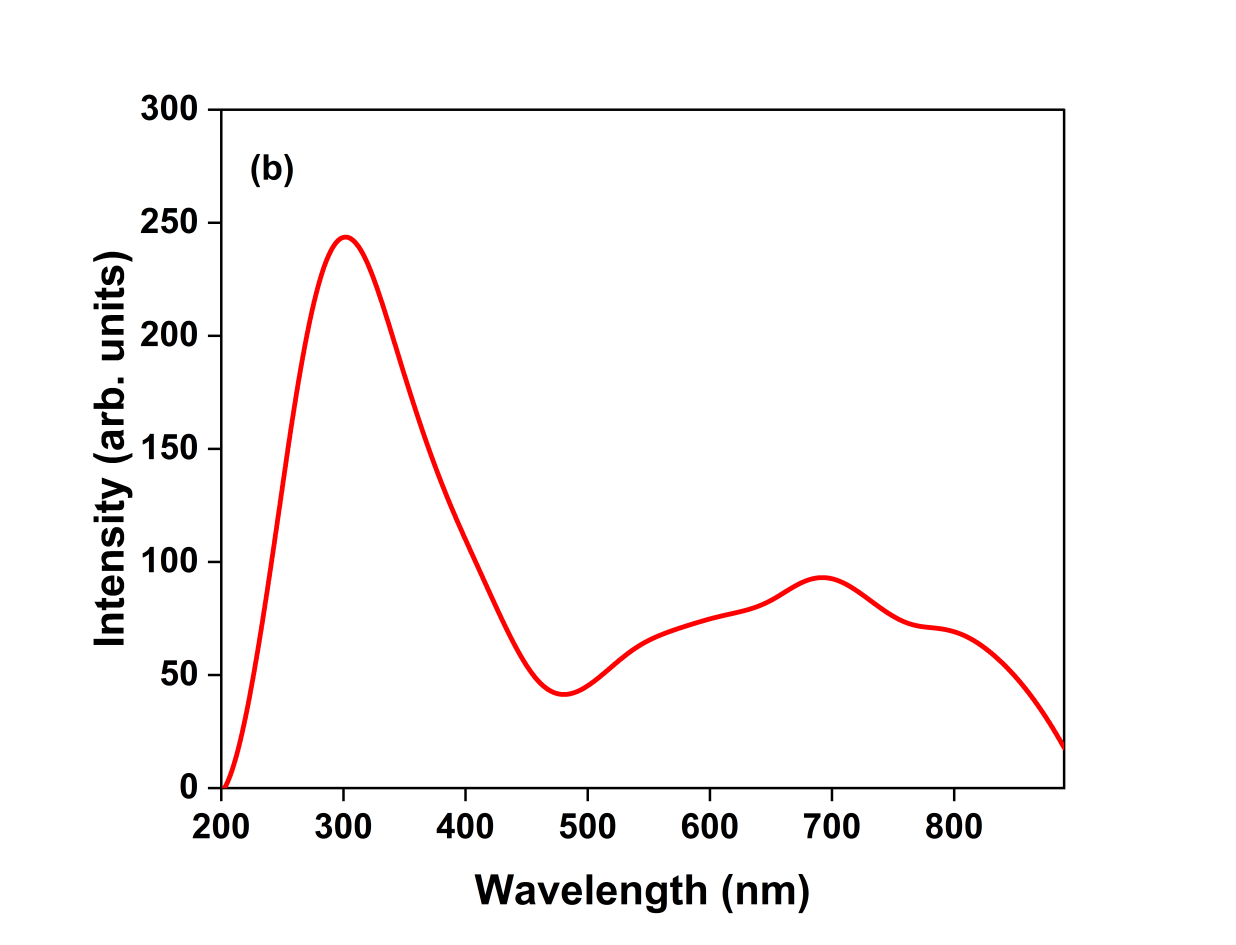


**(a)**

**Fig. S2: a)UV-Visible spectrum of Ag-HA nanocomposite to virgin HA, and b) photolumensence analysis of Ag-HA nanocomposite.**

**
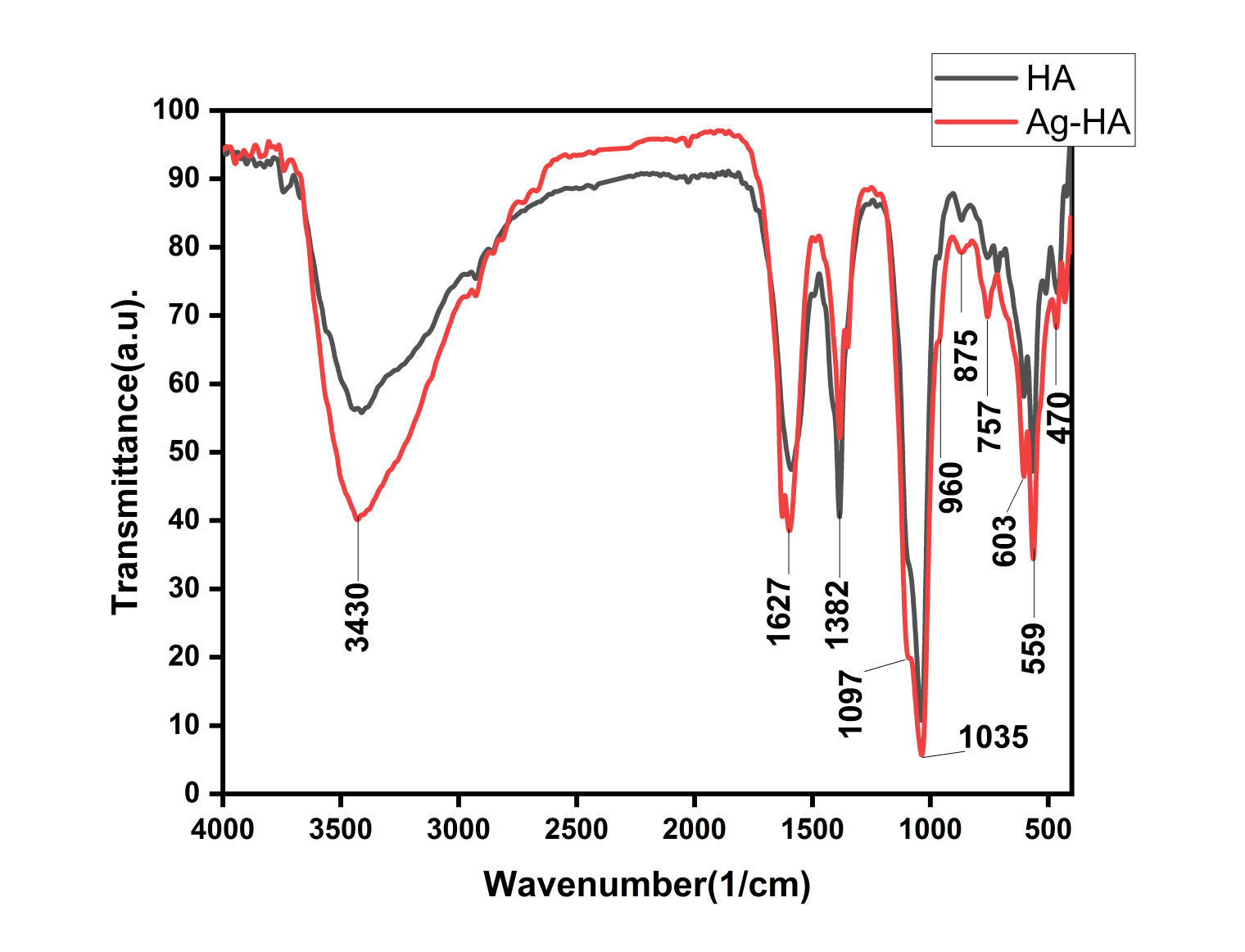
**

**Fig. S3: FTIR spectra of HA and Ag-HA**


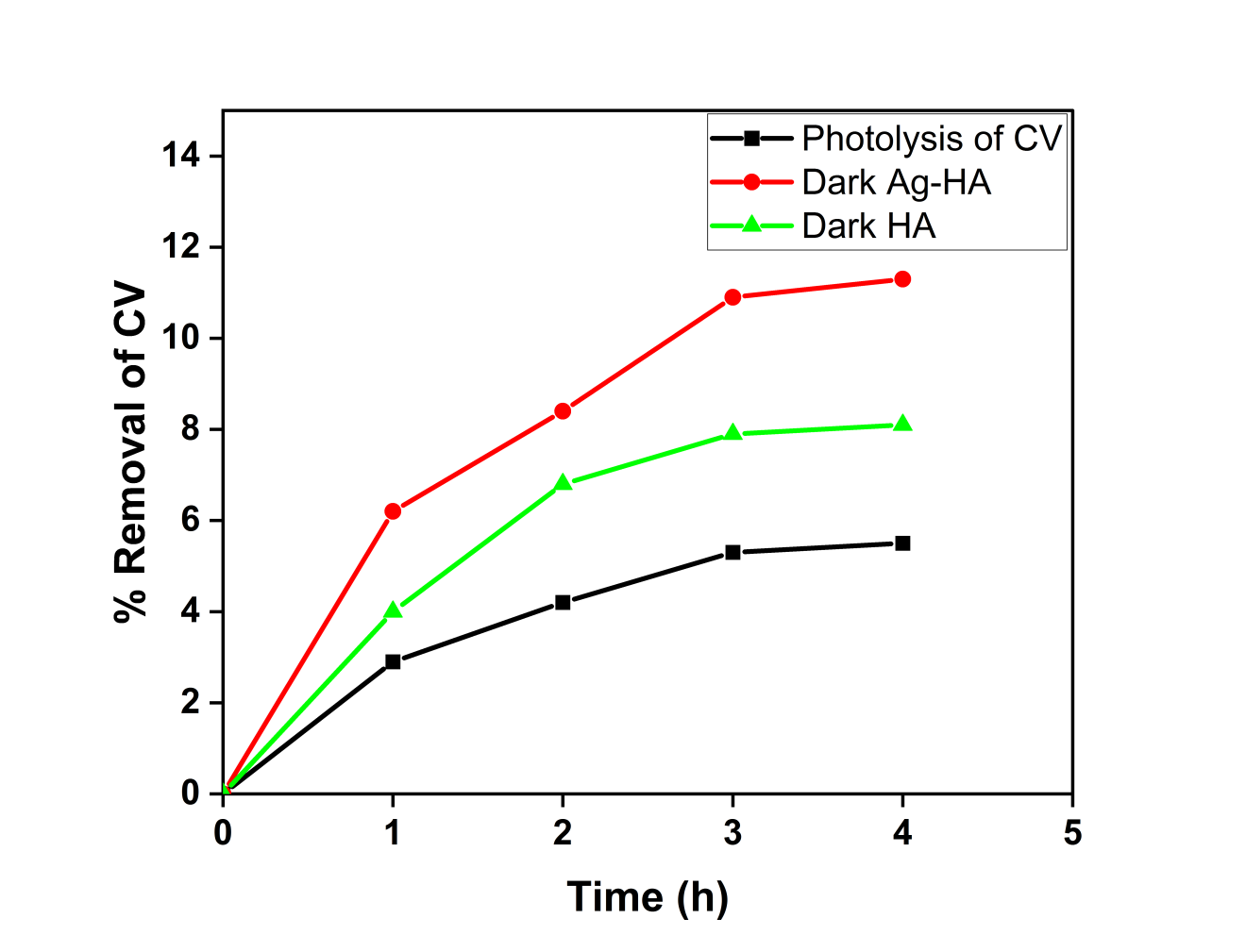


**Fig. S4: Removal of CV within 4 hours due to photolysis (with not catalyst ) (Blackline), adsorption activity of HA (Green line), and adsorption activity of Ag- HA (Redline).**


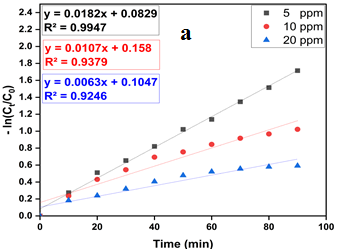


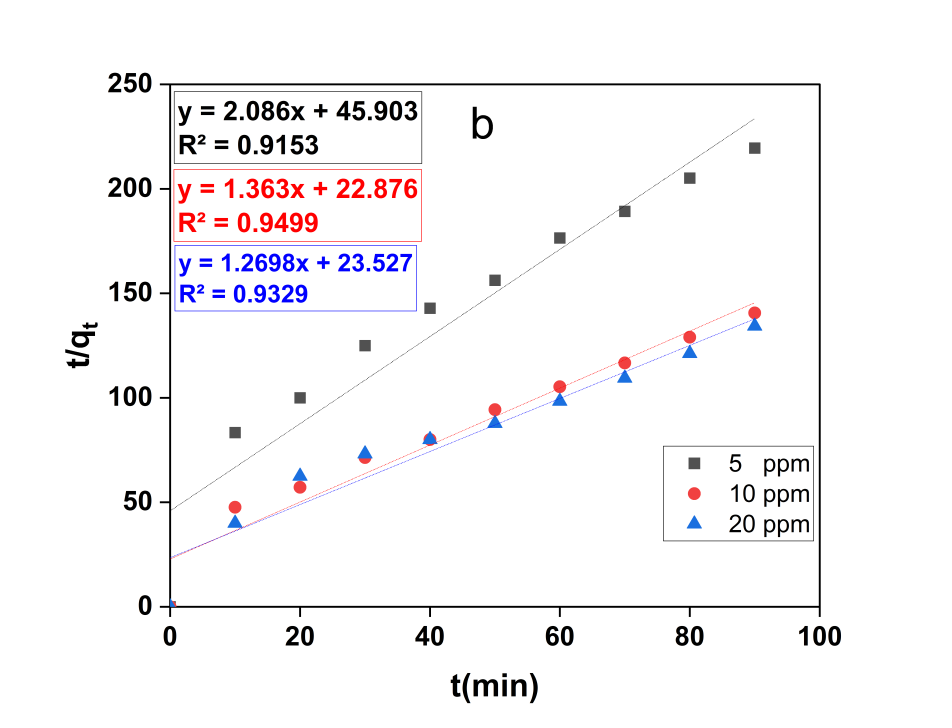


**Fig. S5: Kinetics plots for linear fitting of data obtained from (a) pseudo-first-order reaction model and (b) pseudo-second-order reaction model for Crystal Violet degradation under UV light irradiation and 10 mg catalyst, 25 ^o^C, 50 mL of 5, 10, and 20 mg /L dye concentration.**


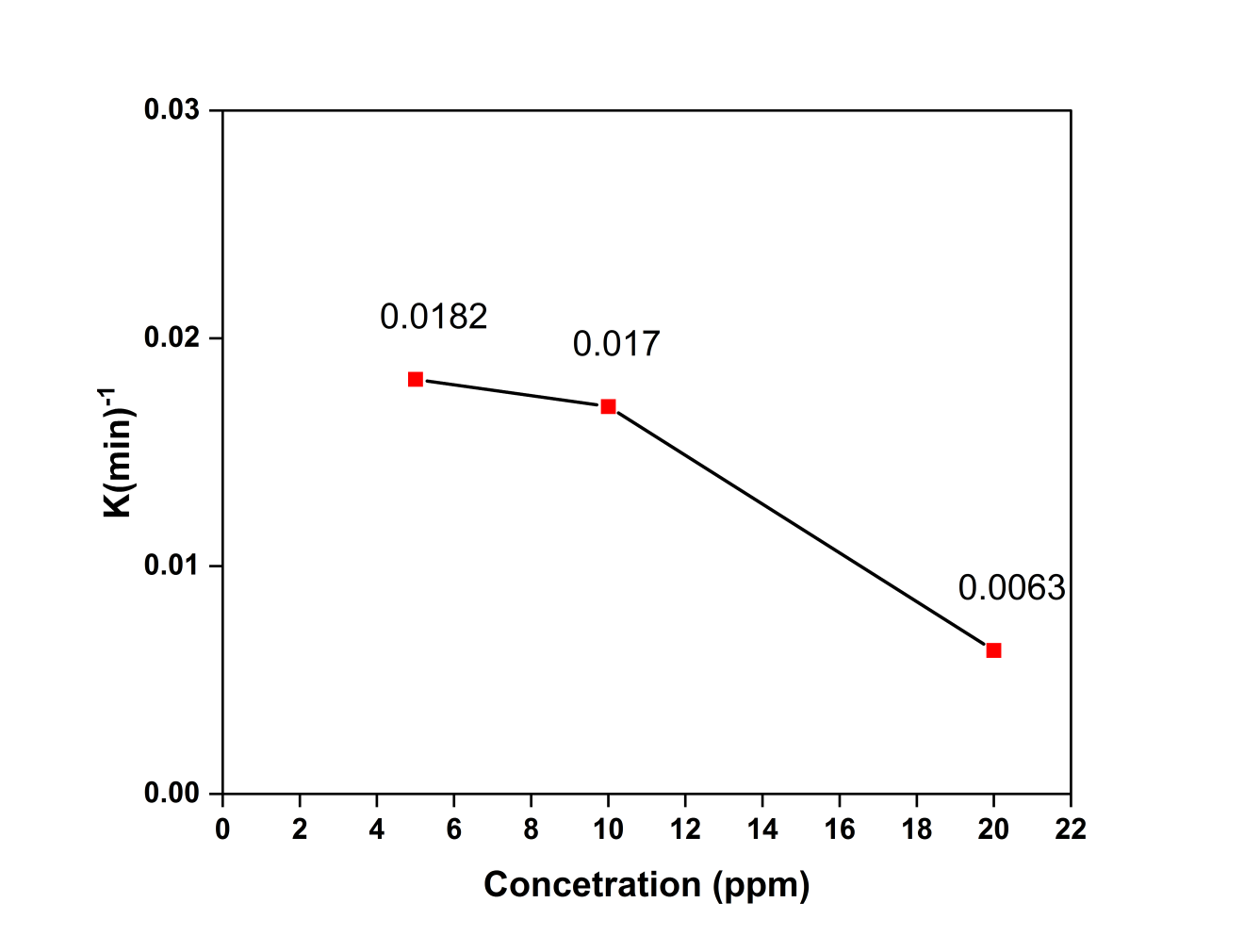


**Fig. S6: The relation of apparent pseudo-first-order rate constants vs. initial concentration of CV.**
